# Mortality from motorcycle crashes: the baby-boomer cohort effect

**DOI:** 10.1186/s40621-016-0083-6

**Published:** 2016-08-09

**Authors:** Victor Puac-Polanco, Katherine M. Keyes, Guohua Li

**Affiliations:** 1 Epidemiology Department, Columbia University in the City of New York, New York, NY USA; 2 Departments of Anesthesiology and Epidemiology, Columbia University in the City of New York, New York, NY USA

**Keywords:** Baby-boomers, Cohort effect, Crashes, Mortality, Motorcycle

## Abstract

**Background:**

Motorcyclists are known to be at substantially higher risk per mile traveled of dying from crashes than car occupants. In 2014, motorcycling made up less than 1 % of person-miles traveled but 13 % of the total mortality from motor-vehicle crashes in the United States. We assessed the cohort effect of the baby-boomers (i.e., those born between 1946 and 1964) in motorcycle crash mortality from 1975 to 2014 in the United States.

**Methods:**

Using mortality data for motorcycle occupants from the Fatality Analysis Reporting System, we performed an age-period-cohort analysis using the multiphase method and the intrinsic estimator method.

**Results:**

Baby-boomers experienced the highest mortality rates from motorcycle crashes at age 20-24 years and continued to experience excess mortality after age 40 years. After removing the effects of age and period, the estimated mortality risk from motorcycle crashes for baby-boomers was 48 % higher than that of the referent cohort (those born between 1930 and 1934, rate ratio 1.48; 95 % CI: 1.01, 2.18). Results from the multiphase method and the intrinsic estimator method were consistent.

**Conclusions:**

The baby-boomers have experienced significantly higher mortality from motorcycle crashes than other birth cohorts. To reduce motorcycle crash mortality, intervention programs specifically tailored for the baby-boomer generation are warranted.

**Electronic supplementary material:**

The online version of this article (doi:10.1186/s40621-016-0083-6) contains supplementary material, which is available to authorized users.

## Background

The morbidity and mortality from traffic injuries worldwide remain an important public health problem (Bandi et al. 2015). Vulnerable road users such as pedestrians, cyclists and motorcyclists continue to be a concern in many countries (ITF 2013; WHO 2015). Globally, motorcyclists account for nearly a quarter of all road traffic deaths (WHO 2015). In 2014, in the United States motorcyclists accounted for less than 1 % of person-miles traveled, but more than 13 % of the total mortality from motor vehicle crashes (FHWA 2015). The proportionate mortality of motorcyclists has almost doubled in the past decade (ITF 2013; NHTSA 2015). The increase in motorcycle crash mortality is due in part to the increased number of motorcycles on public roads; the number of registered motorcycles in the United States increased 116 % from 3.8 million in 1998 to 8.4 million in 2013 (FHWA 2015; US DOT 2015). Furthermore, the increase in motorcycle ownerships appeared to be driven largely by those aged 40 years and over (AMA 2015; FHWA 2015; US DOT 2015). In a 2009 report from the United States Department of Transportation the median age of motorcycle owners increased from 27 years in 1985 to 41 years in 2003 and to 48 years in 2012 (Morris 2009; Shankar and Varghese 2006). The aging of motorcyclists is also reflected in the crash mortality data. In 1982, riders aged 50 years and older accounted for 3 % of all fatally injured motorcyclists, which increased to 13 % in 1997 and 34 % in 2013 (IIHS 2014).

The epidemiologic patterns described above are indicative of a possible cohort effect in motorcycle crash mortality. Cohort effects arise when a specific age group in a particular time period experiences a different risk of morbidity or mortality than other age groups in the same time period. Elucidating cohort effects may help identify the population group at excess risk and develop intervention programs specially tailored for the target population group. Therefore, we performed an age-period-cohort analysis to assess whether the baby-boomer generation experienced a significantly greater mortality from motorcycle crashes.

## Methods

We obtained data on motorcycle traffic casualties from the Fatality Analysis Reporting System -FARS- (NHTSA 2015). This publicly available database, created in 1975, is a nationwide census of fatal motor vehicle crashes that occurred on public roads in the United States (all 50 states, the District of Columbia, and Puerto Rico). FARS contains data from police reports, death certificates, state vehicle registration files, coroner/medical examiner reports, state driver licensing files, hospital medical reports, state highway department data, emergency medical services reports, vital statistics and other state records. The FARS data is organized in three main sub-data sets: person, crash, and vehicle. For this analysis, we used the person data file that contains information for each motorist involved in a crash with details about age, sex, person type (rider or passenger), location and time of the crash, and time of death.

This study included all motorcycle occupants aged 15 to 84 years recorded in FARS who died within 30 days of the crash while traveling on a roadway customarily open to the public in the 50 states and the District of Columbia from 1975 to 2014. The selection of fatalities that occurred within 30 days is based on the fact that these deaths not only could be directly linked as consequences of the crash but also because the data base uses the same time frame (NHTSA 2015). We excluded pedestrians and occupants of non-motorcycle vehicles killed in crashes.

We began the analysis with arranging the data into a contingency table. We grouped the subjects into 15 five-year age groups (from 15–19 years through 80–84 years) and 8 five-year time periods (from 1975–1979 through 2010–2014) (Table 1). We used US Census Bureau data to create the annualized mortality rates by age group, expressed as the number of deaths per 100,000 persons in the United States (US CENSUS BUREAU 2015). This arrangement allows for following each of the 21 birth cohorts as they aged through each of the 8 time periods. For example, in Table 1, individuals born in 1960–1964 were aged 15–19 in 1975–1979 and had a mortality rate of 4.63 per 100,000. This same birth cohort was aged 20–24 in 1980–1984 and had a mortality rate of 6.53 per 100,000. This diagonal pattern enabled us to identify mortality rates for each birth cohort. Also, it is important to mention that for the youngest and oldest birth cohorts located at the extremes of the table, there is limited number of data points. For instance, we only have one data point for individuals born in 1995–1999; their mortality rate was 0.57 per 100,000 in 2010–2014 at 15–19 years old.

**Table 1:** Age-Period Contingency Table for Mortality Rate Per 100,000 by Age (row) and Period (column) in the United States, 1975–2014

	1975–1979	1980–1984	1985–1989	1990–1994	1995–1999	2000–2004	2005–2009	2010–2014
15–19	4.63	4.11	3.46	1.66	0.72	0.78	0.83	0.57
20–24	6.44	6.53	5.41	3.28	2.18	2.38	3.15	2.36
25–29	4.00	4.59	3.77	2.32	1.79	2.17	2.63	2.39
30–34	2.36	2.86	2.41	1.76	1.40	1.91	2.34	1.93
35–39	1.47	1.84	1.58	1.36	1.17	1.76	2.37	1.95
40–44	0.87	1.26	1.15	0.97	1.21	1.84	2.40	2.03
45–49	0.55	0.84	0.72	0.74	0.94	1.75	2.39	2.10
50–54	0.37	0.47	0.48	0.53	0.78	1.60	2.34	2.25
55–59	0.26	0.31	0.35	0.36	0.55	1.20	1.99	2.04
60–64	0.17	0.26	0.22	0.19	0.31	0.70	1.49	1.72
65–69	0.10	0.18	0.13	0.13	0.21	0.48	0.94	1.26
70–74	0.08	0.11	0.10	0.12	0.10	0.26	0.55	0.76
75–79	0.06	0.07	0.04	0.06	0.08	0.12	0.31	0.48
80–84	0.02	0.04	0.05	0.02	0.05	0.09	0.11	0.22

To assess how the process of aging, overall secular trends, and the year of birth influences motorcycle crashes, we need to partition the variance in observed rates over time into age, period, and cohort effects (Browning et al. 2012). However, the linear effects of age, period, and cohort cannot be uniquely identified simultaneously because of the collinearity among the three variables (cohort = period –age) (Keyes and Li 2010, 2014; Tu et al. 2012; Yang et al. 2008). Different methods have been proposed to deal with this identification problem (Keyes and Li 2010, 2014; Keyes et al. 2010; Tu et al. 2012; Yang et al. 2008). In this study, we based our primary analysis on the multiphase method articulated by Keyes and Li (2010) and corroborated the results using the intrinsic estimator method developed by Yang et al. (2004). The first step of the multiphase method is a graphical presentation and inspection of the data from the age-period contingency table. This graphical presentation allows us to examine any unusual pattern among the different birth cohorts. The second step involves a non-parametric median polish analysis to remove the additive effects of age (row) and period (column). Using the contingency table described above we first log-transformed each rate and then we iteratively subtracted the median value of each row and column. The median polish helps isolate the cohort effect from the additive age and period effects in the contingency table data. Finally, a simple linear regression of residuals from the median polish analysis on the cohort identifier is performed to quantify the cohort effects (Keyes and Li 2010). We chose the 1930–1934 birth cohort as the reference cohort because it has the same number of data points as our baby-boomer sub-cohorts (1945–1949, 1950–1954, 1955–1959, and 1960–1964) and its time distance by 10 years from the first baby-boomer sub-cohort. The multiphase method is described in detail elsewhere (Keyes and Li 2010, 2014). To corroborate the results from the multiphase method, we analyzed the data using the intrinsic estimator method, which allows to estimate unique parameters for age, period, and cohort by removing the influence of the design matrix on coefficient estimates (Yang et al. 2004; Yang et al. 2008). This estimable functions yield robust estimates of disease trends by age, period, and cohort and uniquely determines the coefficient estimates (Yang et al. 2004). STATA V.12.1 and Microsoft Excel 2010 were used for the analysis and graphics (Microsoft 2010; StataCorp 2015).

## Results

In the 50 states and the District of Columbia from 1975 to 2014 there were a total of 1,513,937 fatal motor vehicle crashes recorded in the FARS. Motorcyclists meeting our case definition were involved in 138,535 (9.2 %) of these crashes, with 144,151 fatalities. Of them, 91.4 % of the subjects were male and 54.5 % were baby-boomers. Of the 131,814 male motorcyclist fatalities, 96.9 % were drivers. In comparison, 73.5 % of the 12,337 female motorcycle fatalities were passengers. From 1975 to 2014, annual mortality rates from motorcycle crashes per 100,000 population decreased 10.7 %. We also assessed for homogeneity of demographic characteristics among the four baby-boomers’ sub-cohorts. There was significant heterogeneity by gender, ethnicity, race and alcohol tests by birth sub-cohort (Pearson Chi^2^ < 0.001).

**Fig. 1:**
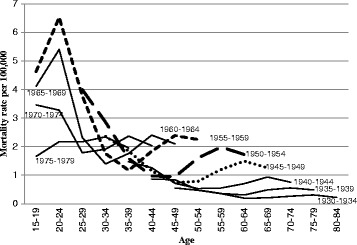
Age-Specific Motorcycle Mortality Rate by Birth Cohort in the United States, 1975–2014

The graphical presentation of the data suggested a polynomial age and period effect overall, and a moderate cohort effect among the baby-boomer sub-cohorts (Fig. 1 & Additional file 1: Figure S1). The increase in mortality rates was present across these sub-cohorts of baby-boomers as they aged. At age 20–24 years, the sub-cohorts born between 1955–1959 and 1960–1964 had the highest mortality rate compared to later birth cohorts (Fig. 1). These baby-boomers appeared to have increased mortality rates at older ages as well. For instance, at age 50–54 years, the sub-cohort of 1955–1959 had more than four times the mortality rate compared to those born in 1940–1944 (Fig. 1 and Table 1).

A linear regression of median polish residuals on cohort category quantified the magnitude of the cohort effect (Additional file 2: Table S1). The baby-boomer sub-cohorts born between 1955–1959 and 1960–1964 had statistically significantly higher mortality rate ratios, 1.48 (95 % Confidence interval: 1.01, 2.18) and 1.47 (95 % Confidence interval: 1.00, 2.17) respectively, compared to the reference cohort; whereas younger cohorts, born after 1980 through 1999, had significant lower mortality rate ratios when compared to the reference cohort (Table 2).

**Table 2:** Estimated rate ratios and 95 % confidence intervals for the cohort effects on motorcycle mortality rate per 100,000 people in the United States, 1975–2014

Birth cohort	Rate ratio	95 % Confidence interval
1895–1899	0.66	0.29, 1.50
1900–1904	0.95	0.51, 1.75
1905–1909	0.97	0.58, 1.64
1910–1914	0.69	0.43, 1.11
1915–1919	0.91	0.59, 1.42
1920–1924	0.92	0.60, 1.39
1925–1929	0.78	0.53, 1.17
1930–1934	1.00	Reference
1935–1939	1.15	0.78, 1.69
1940–1944	1.27	0.86, 1.87
1945–1949	1.43	0.97, 2.10
1950–1954	1.47	1.00, 2.16
1955–1959	1.48*	1.01, 2.18
1960–1964	1.47*	1.00, 2.17
1965–1969	1.17	0.79, 1.75
1970–1974	0.96	0.63, 1.46
1975–1979	0.69	0.45, 1.08
1980–1984	0.46*	0.29, 0.74
1985–1989	0.39*	0.23, 0.65
1990–1994	0.28*	0.15, 0.51
1995–1999	0.17*	0.07, 0.38

* = *P* < 0.05

A stratified analysis by gender showed that the cohort effect ascribed to baby-boomers was stronger in women than in men. The female baby-boomer sub-cohorts of 1955–1959 and 1960–1964 had 1.91 times (95 % Confidence interval: 1.08, 3.38) and 1.94 times the mortality rate (95 % Confidence interval: 1.10, 3.44), respectively, compared to the reference cohort (Table 3).

**Table 3:** Estimated rate ratios and 95 % confidence intervals by gender for the cohort effects on motorcycle mortality rate per 100,000 United States, 1975–2014

	Males		Females	
Birth cohort	Rate ratio	95 % CI^a^	Rate ratio	95 % CI^a^
1895–1899	0.67	0.30, 1.49	1.24	0.37, 4.17
1900–1904	0.95	0.53, 1.72	1.46	0.59, 3.61
1905–1909	1.01	0.61, 1.69	0.39*	0.18, 0.85
1910–1914	0.69	0.43, 1.09	0.78	0.39, 1.57
1915–1919	0.90	0.59, 1.38	1.11	0.58, 2.12
1920–1924	0.91	0.61, 1.36	0.93	0.50, 1.73
1925–1929	0.77	0.52, 1.14	1.14	0.63, 2.06
1930–1934	1.00	Reference	1.00	Reference
1935–1939	1.14	0.78, 1.65	1.54	0.87, 2.72
1940–1944	1.27	0.87, 1.85	1.54	0.87, 2.72
1945–1949	1.44	0.99, 2.10	1.61	0.91, 2.85
1950–1954	1.49*	1.03, 2.17	1.65	0.93, 2.92
1955–1959	1.49*	1.02, 2.16	1.91*	1.08, 3.38
1960–1964	1.48*	1.02, 2.15	1.94*	1.10, 3.44
1965–1969	1.18	0.80, 1.74	1.53	0.85, 2.76
1970–1974	0.98	0.66, 1.47	1.03	0.56, 1.91
1975–1979	0.73	0.47, 1.11	0.63	0.33, 1.22
1980–1984	0.49*	0.31, 0.77	0.44*	0.22, 0.88
1985–1989	0.41*	0.25, 0.68	0.31*	0.14, 0.67
1990–1994	0.29*	0.16, 0.52	0.28*	0.11, 0.68
1995–1999	0.18*	0.08, 0.39	0.14*	0.04, 0.48

* = *P* value < 0.05

^a^CI, Confidence Interval

Results from the intrinsic estimator method showed a similar cohort effect pattern (Additional file 3: Figure S2), a significant higher risk for the 15–39 age groups (Additional file 4: Figure S3) and an upwards period effect since 1995 (Additional file 5: Figure S4).

## Discussion

In this study we used the multiphase method to assess the cohort effect of the baby-boomer generation in motorcycle crash mortality in the United States from 1975 to 2014. Our results indicate that after removing the additive effects of age and period, the 1955–1959 and 1960–1964 birth sub-cohorts had experienced significantly higher mortality from motorcycle crashes than those born between 1930–1934.

Similar findings were reported by Langley et al. (2013) that the 1949–1958, 1954–1963, 1959–1968 and 1964–1973 birth cohorts in New Zealand had an elevated motorcycle crash injury risk relative to the 1944–1953 birth cohort. However, our results were specific to fatalities in two sub-cohorts of the baby-boomers cohort, while Langley and colleagues reported an increased motorcycle injury (fatal and non-fatal) risk compared to other birth cohorts (Langley et al. 2013). Our analysis also suggested a protective cohort effect for cohorts born after 1980 when compared to the reference cohort. However, these results need to be interpreted with caution as the contingency table provides fewer data points for oldest and youngest cohorts located at the extremes of the table.

The excess mortality risk from motorcycle crashes in baby-boomers is likely a function of increased exposure to motorcycling. Many baby-boomers have experienced characteristic changes of their preferred mode of transportation. The oil embargo of the 1970s, which brought price controls, rationing, and a quadrupling of gas prices in just a few months, prompted baby-boomers to alternate forms of transportation, including motorcycles, when they were young (McGuckin and Lynott 2012). Recently, ownership and mortality data suggest that there is renewed interest among baby-boomers in driving motorcycles (Morris 2009; Shankar and Varghese 2006). Baby-boomers as a whole also have a higher level of disposable income than other birth cohorts, which enables them to afford the motorcycle ownership and embrace the motorcycle culture at the retirement age. In 2013, baby-boomers aged between 55–64 years old had a mean income of $53,000 while people aged 55–64 years back in 1993 had a mean income close to $40,000 (2014 CPI-U-RS adjusted dollars) (US CENSUS BUREAU 2014). This economic factor is used by the motorcycle industry to target baby-boomers for expanding the motorcycle sales (Maynard 2002; Sizemore 2013).

Our analysis also revealed that the baby-boomer effect in motorcycle crash mortality appeared to be more pronounced in women than in men. The sex-cohort interaction on motorcycle crash mortality is likely multifactorial. It is plausible that exposure to motorcycling in female baby-boomers might be more prevalent than in other female cohorts and that the difference in exposure prevalence between baby-boomers and other cohorts might be greater in women than in men.

There are several notable limitations with our study. First, we were unable to account for miles travelled per driver by age group. The cohort effect found in our analysis might be explained by baby-boomers consistently traveling more miles per driver when compared to other birth cohorts (McGuckin and Lynott 2012). Second, our analysis did not take into consideration any risk-taking behavior, such as alcohol and drug use, speeding, and not wearing helmets. Future research may help determine the relative contributions of exposure to motorcycling and specific risk-taking behaviors to the observed cohort effect in motorcycle mortality ascribed to baby-boomers.

## Conclusion

Nevertheless, our study suggests that baby-boomers are at heightened risk of motorcycle mortality and that it is necessary to develop intervention programs tailored for baby-boomers who are between 52 to 70 years of age in 2016. Implementation of mandatory courses for re-entry riders and continuing road safety training for older adult riders may help recognize and mitigate risks associated with cognitive and health declines during the process of aging, such as increased medication use, frailty and fragility, and reduce motorcycle crash mortality.

## Abbreviations

CPI-U-RS, Consumer Price Index Research Series Using Current Methods; FARS, Fatality Analysis Reporting System

## Additional files

Additional file 1: Figure S1. Baby-boomers graphical trend compared to other birth cohorts in the United States, 1975–2014 (Red solid diamonds represent mortality rates for baby-boomers as the aged from 1975 to 2014; blue solid squares represent other birth cohorts). (DOCX 160 kb) Additional file 2: Table S1. Residuals from median polish^a^ based on mortality rates in the United States, 1975–2014. (DOC 38 kb) Additional file 3: Figure S2. Intrinsic Estimator Cohort effect in the United States, 1975–2014. (DOCX 22 kb) Additional file 4: Figure S3. Intrinsic Estimator Age effect in the United States, 1975–2014. (DOCX 21 kb) Additional file 5: Figure S4. Intrinsic Estimator Period effect in the United States, 1975–2014. (DOCX 21 kb)
