# Supplementary material for: Mortality from motorcycle crashes: the baby-boomer cohort effect
Source: Inj Epidemiol. 2016 Aug 9;3(1):19. doi: 10.1186/s40621-016-0083-6 (PMC4978754; doi:10.1186/s40621-016-0083-6)
Supplement: Additional file 1: Figure S1. — Baby-boomers graphical trend compared to other birth cohorts in the United States, 1975–2014 (Red solid diamonds represent mortality rates for baby-boomers as the aged from 1975 to 2014; blue solid squares represent other birth cohorts). (DOCX 160 kb) [file 40621_2016_83_MOESM1_ESM.docx]

| **Additional file 1: Figure S1.** Baby-boomers graphical trend compared to other birth cohorts  in the United States, 1975-2014 ^a^ | | |
| --- | --- | --- |
| 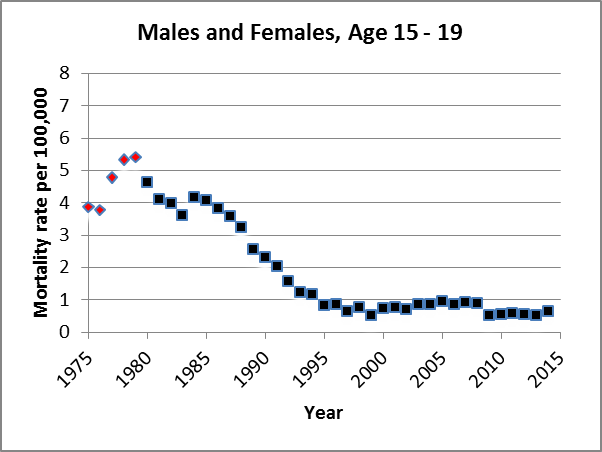 | 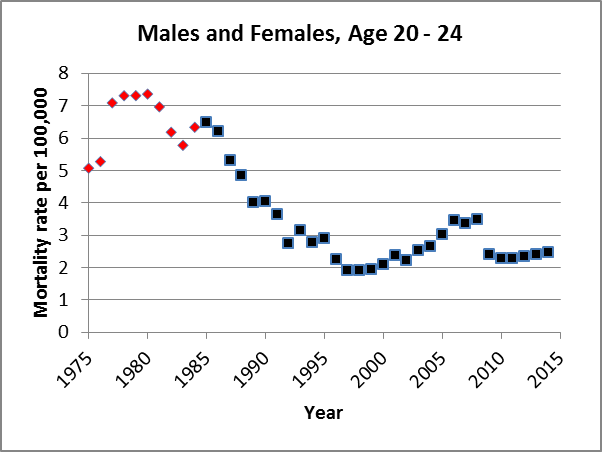 | 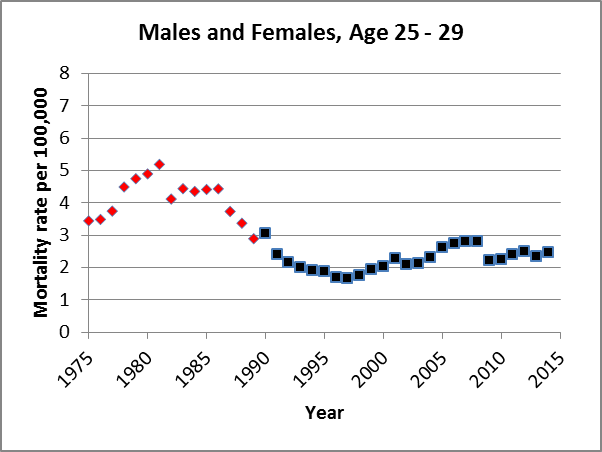 |
| 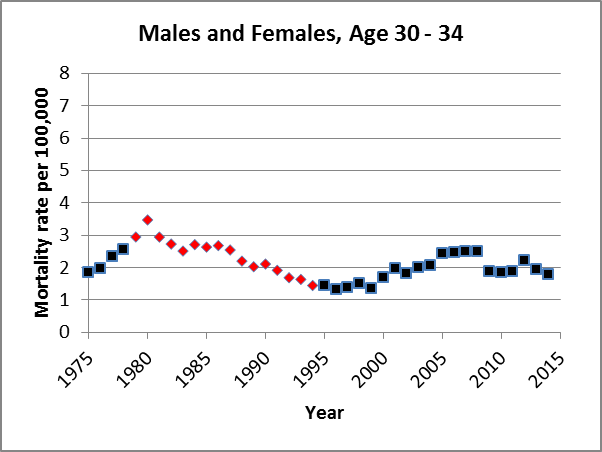 | 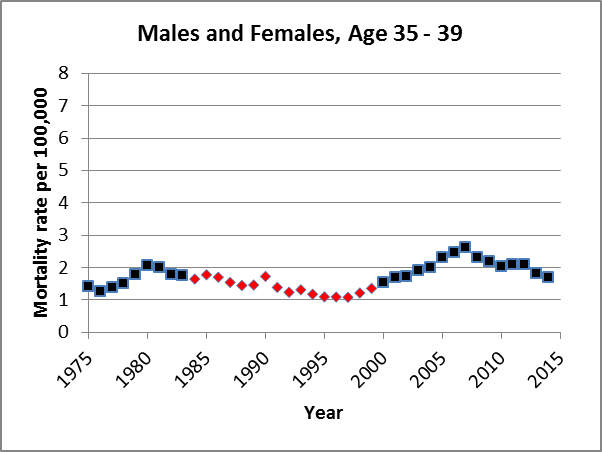 | 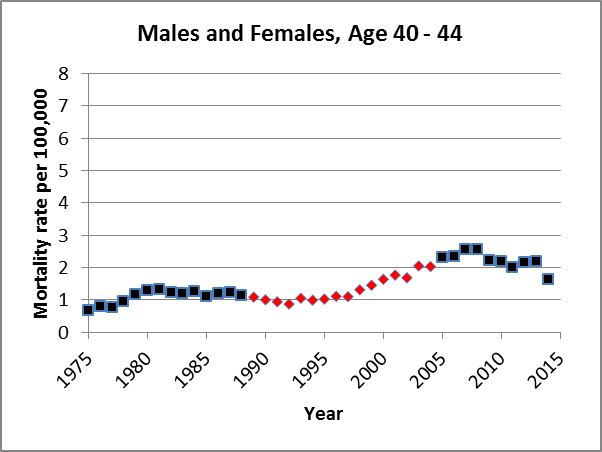 |
| 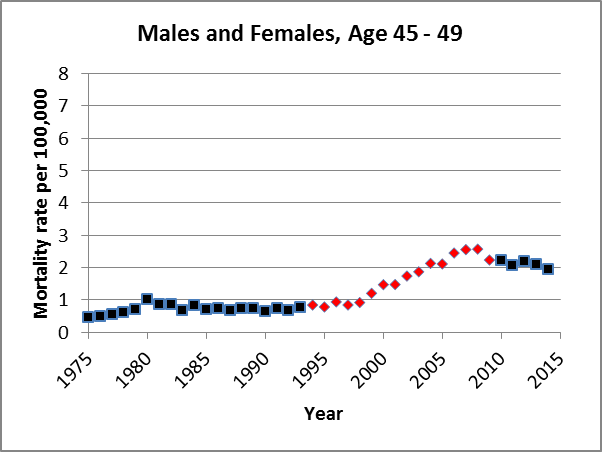 | 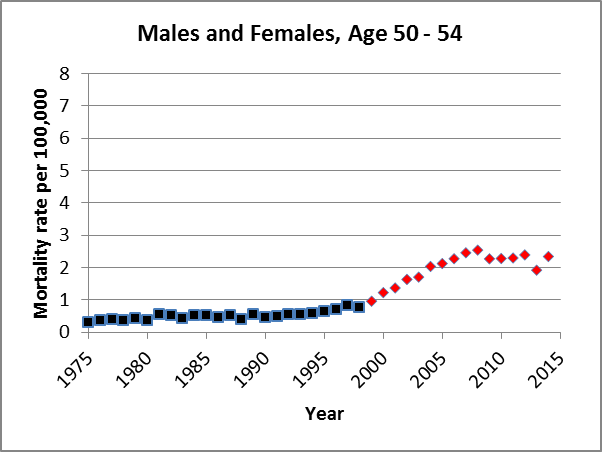 | 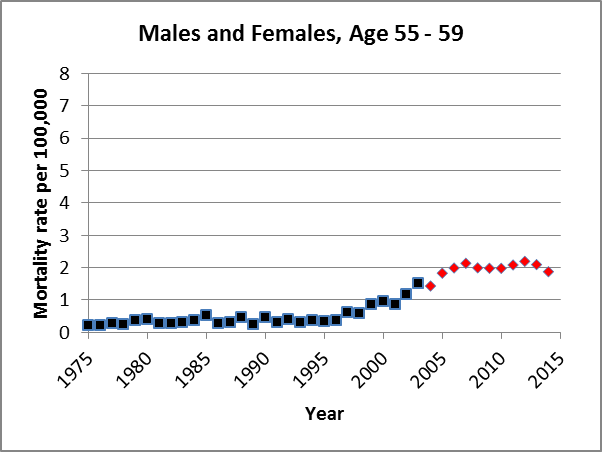 |

^a^ Red solid diamonds represent mortality rates for baby-boomers as the aged from 1975 to 2014; blue solid squares represent other birth cohorts
