# Supplementary material for: Mortality from motorcycle crashes: the baby-boomer cohort effect
Source: Inj Epidemiol. 2016 Aug 9;3(1):19. doi: 10.1186/s40621-016-0083-6 (PMC4978754; doi:10.1186/s40621-016-0083-6)
Supplement: Additional file 2: Table S1. — Residuals from median polisha based on mortality rates in the United States, 1975–2014. (DOC 38 kb) [file 40621_2016_83_MOESM2_ESM.doc]

**Additional file 2: Table S1.** Residuals from median polish^a^ based on mortality rates

in the United States, 1975-2014

|  | **1975-1979** | **1980-1984** | **1985-1989** | **1990-1994** | **1995-1999** | **2000-2004** | **2005-2009** | **2010-2014** |
| --- | --- | --- | --- | --- | --- | --- | --- | --- |
| **15-19** | 1.75 | 1.32 | 1.20 | 0.53 | -0.53 | -0.97 | -1.28 | -1.73 |
| **20-24** | 1.19 | 0.89 | 0.75 | 0.32 | -0.32 | -0.75 | -0.84 | -1.21 |
| **25-29** | 0.98 | 0.81 | 0.66 | 0.24 | -0.24 | -0.57 | -0.75 | -0.93 |
| **30-34** | 0.72 | 0.60 | 0.48 | 0.23 | -0.23 | -0.44 | -0.60 | -0.88 |
| **35-39** | 0.46 | 0.37 | 0.27 | 0.19 | -0.19 | -0.30 | -0.37 | -0.65 |
| **40-44** | 0.09 | 0.14 | 0.10 | 0.00 | 0.00 | -0.11 | -0.21 | -0.46 |
| **45-49** | -0.10 | 0.00 | -0.10 | 0.00 | 0.02 | 0.11 | 0.05 | -0.16 |
| **50-54** | -0.24 | -0.31 | -0.24 | -0.08 | 0.08 | 0.28 | 0.29 | 0.16 |
| **55-59** | -0.23 | -0.36 | -0.19 | -0.10 | 0.10 | 0.36 | 0.49 | 0.43 |
| **60-64** | -0.14 | -0.02 | -0.13 | -0.21 | 0.02 | 0.34 | 0.72 | 0.78 |
| **65-69** | -0.26 | 0.00 | -0.27 | -0.21 | 0.00 | 0.33 | 0.63 | 0.84 |
| **70-74** | -0.09 | -0.08 | -0.14 | 0.08 | -0.30 | 0.15 | 0.52 | 0.75 |
| **75-79** | 0.10 | -0.08 | -0.50 | -0.02 | 0.02 | -0.11 | 0.46 | 0.81 |
| **80-84** | -0.38 | -0.12 | 0.21 | -0.48 | 0.05 | 0.14 | -0.05 | 0.53 |

^a^ Previous to the median polish iterations, we transformed the rates to the natural log scale.
