# Supplementary material for: Mortality from motorcycle crashes: the baby-boomer cohort effect
Source: Inj Epidemiol. 2016 Aug 9;3(1):19. doi: 10.1186/s40621-016-0083-6 (PMC4978754; doi:10.1186/s40621-016-0083-6)

Additional file 5: Figure S4. Intrinsic Estimator Period effect in the United States, 1975-2014

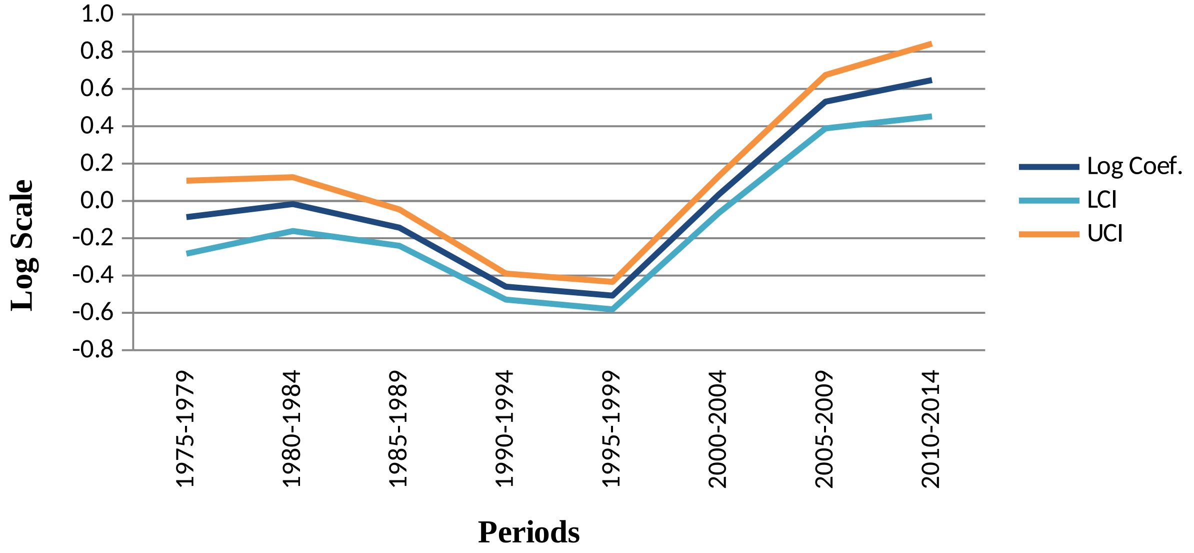

Supplement: Supplementary file 5 [file 40621_2016_83_MOESM5_ESM.docx]
